# Examining the Impact of Certificate of Need Laws on the Utilization and Reimbursement of Cataract Surgeries Among Medicare Beneficiaries

**DOI:** 10.36469/001c.121618

**Published:** 2024-08-13

**Authors:** Alvina Liang, Jennifer L. Lindsey

**Affiliations:** 1 Vanderbilt University School of Medicine, Nashville, Tennessee, USA; 2 Department of Ophthalmology Vanderbilt Eye Institute, Nashville, Tennessee, USA

**Keywords:** certificate of need, cataract surgery, ophthalmology, health policy, government regulation

## Abstract

**Background:** Cataract surgery is an effective and commonly utilized procedure and can significantly improve quality of life and restore economic productivity. Certificate of need (CON) laws aim to regulate healthcare facility expansion and equipment acquisition to curtail costs, enhance quality, and ensure equitable access to care. However, little is known about the impact of CON laws on cataract surgery utilization and reimbursement.

**Objectives:** To compare utilization and reimbursement for non-complex cataract surgery in CON and non-CON states.

**Methods:** This retrospective database review analyzed publicly available data from the Centers for Medicare and Medicaid Services from 2017 to 2021 to identify the Medicare beneficiaries who underwent non-complex cataract surgery using Current Procedural Terminology code 66984 in Medicare outpatient hospitals. Utilization and reimbursement patterns were analyzed in states with and without CON laws using the compound annual growth rate, with reimbursement adjusted by the US Bureau of Labor Statistics Consumer Price Index.

**Results:** The Centers for Medicare and Medicaid Services reported 893 682 non-complex cataract surgeries in the study period; of these, 609 237 were in CON and 280 215 in non-CON states. Inflation-adjusted reimbursement increased in both CON (1.17%) and non-CON (1.83%) states, while the reimbursement in non-CON states was greater than the national average adjusted reimbursement (1.67%). Utilization of non-complex cataract surgery declined during the study period in both CON and non-CON states. A larger decline in utilization was observed in CON states (−7.32%) than in non-CON states (−6.49%). Utilization was slightly higher in non-CON than in CON states for each year except 2019.

**Discussion:** Utilization of non-complex cataract surgery by Medicare beneficiaries declined over the study period in both CON and non-CON states, possibly impacted by the COVID-19 pandemic. Inflation-adjusted reimbursement adjusted for Consumer Price Index increased more in non-CON than CON states, possibly reflecting shifts in market dynamics in CON-regulated states.

**Conclusions:** Surgeons and policymakers should consider the implications of CON laws on the utilization and reimbursement of cataract surgery. Further study is necessary to ascertain whether these trends persist beyond 2021.

## INTRODUCTION

Cataract is a leading cause of reversible blindness and visual impairment worldwide.^1^ A recent study estimated that over 160 million people of working age have significant visual impairment, and employment in this group may be reduced by nearly a third. The annual global cost of lost productivity using gross domestic product (GDP) models may be as large as $322.1 to $518.7 billion, or 0.3% of GDP.^2^ Cataract surgery is a safe and effective cure for visual impairment due to cataract. It is the most commonly performed surgery in ambulatory surgery centers (ASCs) in the United States, accounting for nearly one-fifth of surgical procedures,^3,4^ and traditionally has been among the most common surgeries provided to Medicare enrollees.^5^ It is important to note that cataract surgery for Medicare beneficiaries must meet medical criteria demonstrating necessity of the procedure in order for the medical provider and facility to be reimbursed. A recent meta-analysis of economic evaluations of cataract demonstrated evidence that cataract surgery is more cost-effective than other ophthalmic and non-ophthalmic health interventions, improving both functionality and subjective quality of life.^6^

Certificate of need (CON) laws are intended to help control healthcare costs by avoiding redundant services and ensuring that new projects and capital expenditures for healthcare facilities meet the needs of their community. They are state-level regulatory mechanisms.^7^ The primary purpose of a CON law is to regulate and oversee healthcare facility expansions, medical equipment acquisition, and the establishment of healthcare services. The utilization of CON laws, however, may differ between states.^8^ Some CON laws aim to control healthcare costs by preventing the unnecessary duplication of healthcare facilities and services. By requiring providers to demonstrate a need for new facilities or services, CON laws seek to ensure that resources are allocated efficiently. Other CON laws are utilized to enhance equitable access to care, as states consider whether the proposed healthcare facilities or services will enhance access to care for underserved populations. By evaluating the need for new healthcare facilities and services, CON laws also aim to prevent overutilization of healthcare resources, with goals to help control healthcare expenditure and ensure that services are used appropriately. However, despite the proposed goals of CON laws, it is imperative to investigate the intended and unintended effects of CON laws. Literature has indicated that CON laws are associated with a reduction in the number of hospital beds and a decrease in healthcare expenditures.^9^ Regarding healthcare quality and access, the impact of CON laws varies. Some studies suggest that while CON laws may maintain or improve quality by regulating facility standards, they can also limit access to care in areas where demand for services exceeds supply, such as nursing homes and mental health services.^10,11^ Therefore, while CON laws aim to achieve cost containment and equitable access, their effects on healthcare quality and access need careful consideration and evaluation.

Despite many studies of the economics of cataract surgery, none has directly assessed the impact of CON on the utilization and reimbursement of the procedure. The objective of this study was to compare utilization and reimbursement for non-complex cataract surgery in CON and non-CON states.

## METHODS

A retrospective analysis of publicly available data from the Centers for Medicare and Medicaid Services (CMS) was performed. *Medicare Outpatient Hospitals – by Geography and Service* and *Medicare Outpatient Service Data* reports from 2017 through 2021 were accessed via data.cms.gov. The study was exempt from Institutional Review Board approval, as no identifiable patient data were included. This methodology is consistent with current literature that evaluates the effect of CON on the utilization and reimbursement trends in other simple surgeries.^12,13^ All Medicare outpatient facilities including hospitals and ASCs were included. This study was limited to the most commonly used cataract surgery CPT code, 66984. This procedure is indicated for visually significant cataract that impairs vision and results in a functional deficit for the patient, in the absence of any comorbid factors (eg, poor pupillary dilation) that would make the surgery complex. The query included all available data for all enrollees who underwent non-complex cataract surgery under this code during the period. Data for Maryland were not available. US territories and possessions were excluded, as was the District of Columbia.

**Figure 1.**
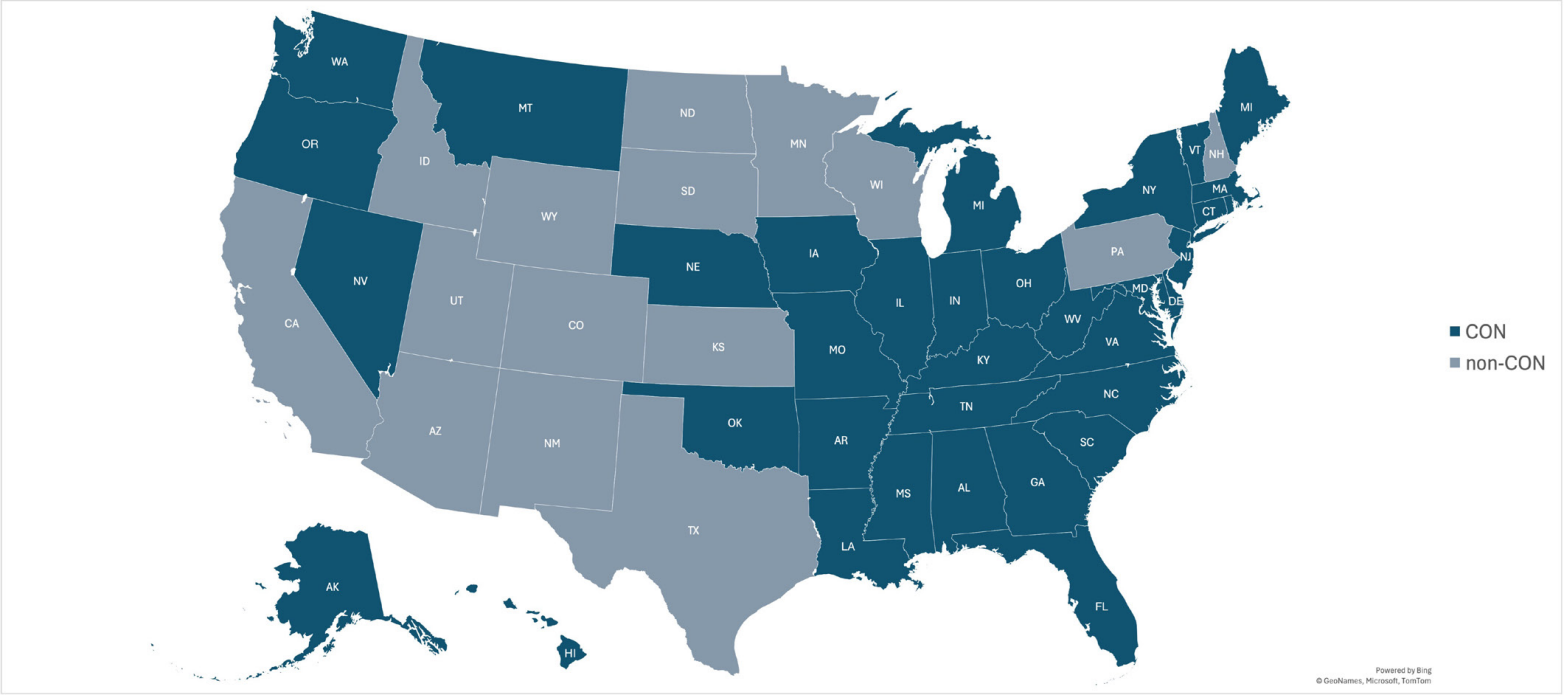
Distribution of CON and Non-CON States in the United States Note: This map shows, by color, CON law states and non-CON states during the study period.

The analysis divided states into those having CON laws and those without. **Figure 1** shows the distribution of CON vs non-CON states on a map of the United States. A list of states in the 2 categories is provided in **Supplemental Table S1**. The number of enrollees who received non-complex cataract surgery in each state was recorded for each of the study years. This was then compared with the total number of enrollees in the state who received any Medicare service in the same year to determine the percent utilization among enrollees in that state. Each year, the average Medicare outpatient hospital/ASC payment was determined by state. Trends in utilization and reimbursement were analyzed. A paired 2-sample *t*-test for means was used to compare the utilization and reimbursement between CON and non-CON states over time. Compound annual growth rate (CAGR) was used to analyze and report relative growth in utilization over the period. Reimbursement growth was adjusted by the United States Bureau of Labor Statistics Consumer Price Index (CPI) calculated over the study period to correct for inflation.^14,15^ The national average percent utilization and average reimbursement were also calculated over the time period, and CON and non-CON trends were compared with each other and the national averages.

## RESULTS

**Figure 2.**
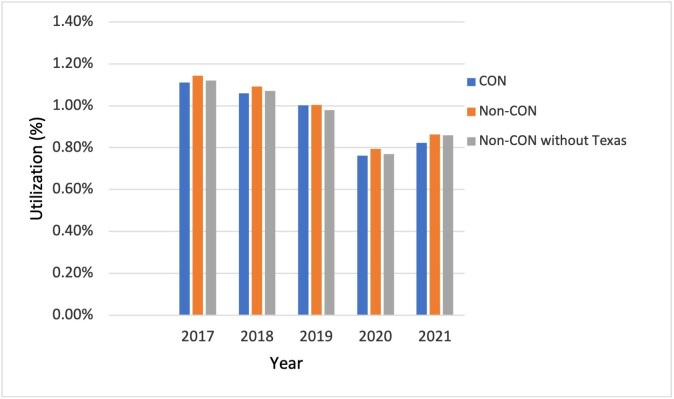
Utilization of Cataract Surgery Among Medicare Beneficiaries Compared with CON states, non-CON states had a significantly higher utilization percentage. When Texas, a highly populated non-CON state, was excluded from the analysis, the difference in utilization between CON and non-CON states was not statistically significant.

CMS reported 893 682 non-complex cataract surgeries in the study period; of these, 609 237 were in CON and 280 215 in non-CON states. Results are shown in **Table 1**. Utilization of non-complex cataract surgery declined during the study period. A larger decline in utilization was observed in CON states (−7.32%) than in non-CON states (−6.49%) (*P* = .015). Utilization was higher in non-CON than in CON states for each year except 2019. **Figure 2** demonstrates the trend in utilization for CON and non-CON states over the study period. A simple paired *t*-test indicates that the average utilization percentage over the 5 years was higher for non-CON states. The difference (non-CON – CON) was 0.00036 (0.00015, 0.00056; *P* = .0087). To examine the possible influence of state-level population size on this difference, additional analyses were conducted excluding Texas, a non-CON state with a large population. This led to a decrease in the difference in average utilization that was not statistically significant (*P* = .21). To further assess this effect, California, another highly populous non-CON state, was excluded. In this scenario, the mean difference in utilization between CON and non-CON states maintained statistical significance (*P* = .0418).

**Table 1.** Utilization and Reimbursement of Non-Complex Cataract Surgery: 2017-2021

	**CON**			**Non-CON**			**National**		
	**Utilization**		**Reimbursement**	**Utilization**		**Reimbursement**	**Utilization**		**Reimbursement**
	**n**	**%**		**n**	**%**		**n**	**%**	
Year									
2021	132 540	0.82	$1608.48	62 965	0.86	$1688.66	197 201	0.83	$1656.42
2020	120 395	0.76	$1563.85	56 014	0.79	$1617.45	176 923	0.76	$1594.55
2019	172 385	1.00	$1458.07	76 400	1.00	$1514.37	249 688	0.99	$1487.82
2018	183 917	1.06	$1464.21	84 836	1.09	$1509.03	269 870	1.05	$1486.62
2017	193 828	1.11	$1408.67	88 085	1.14	$1431.61	282 960	1.12	$1415.86
Total	803 065			368 300			1 176 642		
Average		0.95	$1500.65	0.98		$1552.22		0.95	$1528.25
Average increase/y	−15 322		$49.95	−6280		$64.26	−21 440		$60.14
CAGR	−7.32%		2.69%	−6.49%		3.36%	−6.97%		3.19%
Adjusted for CPI			1.17%			1.83%			1.67%

Abbreviations: CAGR, compound annual growth rate; CON, certificate of need; CPI, Consumer Price Index.

**Figure 3.**
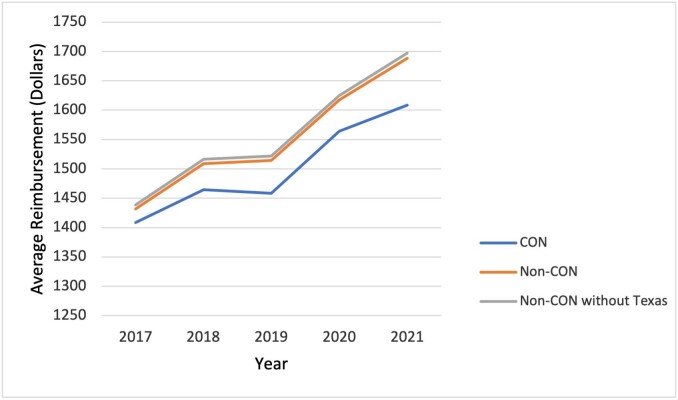
Average Medicare Payment for Cataract Surgery Non-CON states had a higher average reimbursement amount than their CON-state counterparts. When Texas, a highly populated non-CON state, was excluded from the analysis, the difference in average reimbursement between CON and non-CON states remained statistically significant.

Mean outpatient hospital/ASC reimbursement was higher in non-CON states ($1552) than in CON states ($1501), *P* = .005. The highest average reimbursement was seen in non-CON states ($1689). Adjusted reimbursement increased in both CON (1.17%) and non-CON (1.83%) states. The increase in adjusted reimbursement in non-CON states was greater than the national average adjusted reimbursement (1.67%): and that of CON states was lower than the national average. **Figure 3** illustrates the trend in reimbursement in CON and non-CON states over the study period.

## DISCUSSION

We examined the effects of CON laws on the 2 factors that directly contribute to Medicare spending on cataract surgery: the utilization of cataract surgery and Medicare reimbursement. Cataract surgery is the most utilized surgery among Medicare beneficiaries.^16^ The incidence of cataract surgery in the United States increased more than 4-fold between 1980 and 1995, largely due to advances in surgical technique and efficiency.^5^ Growth rates leveled in the late 1990s and early 2000s but have since increased with the overall aging of the US population. Our study showed that during the study period, cataract utilization over the 5 years was statistically higher for non-CON states when the data compared cataract utilization in all non-CON states vs CON states. Variation in state size had an indeterminate effect, as illustrated by the disparate results when Texas or California, each a large-population non-CON state, was excluded from the analysis. When Texas, a non-CON state with a large population and high utilization, was excluded, the difference in utilization between CON and non-CON states narrowed and the *P* value became 0.21. High cataract surgery utilization in Texas was unexpected, as some studies have suggested that Texas is a state that has a higher-than-average travel burden for its patients to receive ophthalmologic care for cataract surgery.^17^

Furthermore, our study found that utilization declined over the study period in both CON and non-CON states. The COVID-19 pandemic dramatically influenced the medical landscape in terms of healthcare utilization in both CON and non-CON states. In ophthalmology, there were sharp decreases in cataract surgical numbers in the United States and elsewhere, due to the closure of clinics and operating rooms for elective surgeries. Ophthalmologic examinations to detect cataracts require close, face-to-face contact between doctor and patient. During the pandemic, many ophthalmology practices were closed completely or operated at significantly reduced capacity, seeing only patients with urgent and emergent conditions. Elective (nonurgent) surgery was discontinued entirely for several months at most hospitals and ASCs. A sharp decline in cataract surgery occurred in 2020 and was sustained into 2021.^18^ The decline in utilization observed in both CON and non-CON states in this study, particularly during 2019-2021, likely reflects the restrictions on elective surgery associated with COVID-19.

The high utilization of cataract surgery by Medicare beneficiaries translates to significant associated costs. Although cataract surgery is highly cost-effective, the sheer volume of surgeries leads to large expenditures of Medicare dollars. Outpatient hospital payments for simple cataract surgery cost the Medicare program over $400 million annually. In this study, reimbursement adjusted for CPI over the study period increased more in non-CON than in CON states, as shown in **Figure 3**. However, in contrast to utilization, the higher reimbursement rate in non-CON states was rather consistent, even when highly populous states such as Texas were excluded from the analysis. Several explanations can explain our result. First, CON laws restrict the expansion of healthcare services and facilities, which can reduce competition between existing providers.^10^ In a less competitive environment, providers may have less incentive to negotiate for higher reimbursement rates with insurance companies or government payers. Moreover, CON laws require approval processes for service expansion, which may limit services and equipment that incorporate higher and newer technology, and further decrease the ability to negotiate for higher reimbursement rates for providers. As technology advances and surgical training becomes more complex and time-intensive, the costs to surgeons and healthcare facilities also increase. Growth in reimbursement that, at a minimum, keeps pace with inflation, is necessary to keep doctors’ offices and ASCs open and able to provide these services.

Health care is not a simple, well-defined commodity, but rather a highly varied personal service. The demand for health care, and quality health care, is highly elastic.^19^ As our population ages and the proportion in the labor force dwindles, available Medicare funds will likely decrease. Cataract surgery is elective. However, if cataracts are untreated and result in significant visual impairment, they can rob patients of their ability to drive, care for themselves, and engage in productive work. In an affluent society like the United States, demand for sight-restoring surgery is unlikely to decline, even when resources are relatively limited. The shift out of larger hospitals and into ASCs, which was dramatic from 2001 to 2014,^4^ represents the effort to make these elective surgeries more efficient and to increase convenience and affordability for patients. Although reimbursement rates for the ASC settings are lower than those for hospitals, the loss is recouped by an increase in volume and efficiency.

The implementation goal of CON law is to curtail duplication and proliferation of healthcare services.^19^ Although CON programs appear to have achieved some benefits, the total costs to impose such programs exceed those benefits by an estimated $302 million a year, with a 1.08 cost-benefit ratio.^8^ In addition, these laws have the (perhaps unintended) consequence of placing the power on the current medical providers in a region to object to the projects of their competitors. There is evidence showing the market entry for new hospitals and nonhospital providers are limited by CON laws, whereas incumbent hospital providers remain largely unaffected. Furthermore, patients who reside in CON states may be induced to travel farther, and more often, for medical care than patients residing in non-CON states. By restricting regional competition from new hospitals, incumbent healthcare providers oftentimes are doing so at the expense of patients.^20^ A similar study in orthopedics found more robust growth and higher reimbursement for cervical discectomy in non-CON states.^13^

This study is limited by the period studied, in which COVID restrictions likely impacted the utilization of elective cataract surgery. However, while utilization of cataract surgeries declined overall, the impact of the COVID pandemic should have impacted the CON and non-CON states to a similar degree. The period was constrained by the public availability of data for the years 2017-2021 only. Another limitation of this study is the limited consideration for extraneous variables and changes within CON and non-CON states during the study period. These changes include the implementation year of CON laws, as well as changes in the regulation of CON within the states during the study period. Further, the data included all outpatient facilities and could not be split into ASCs and other outpatient hospital settings. Data for Maryland were not available. There are many CPT codes for cataract surgery, including codes for complex cases that reimburse at a higher rate. This study focused only on 66984, the code for non-complex cataract surgery. Only Medicare data were analyzed, and there is likely less variability in Medicare payments for similar services across states than might be expected for private insurance payers. For this study’s purposes, Arizona, Minnesota, and Wisconsin were included as non-CON states, although they enforce some restrictions on healthcare service expansions.

Given the added variance in economic factors during COVID-19, further studies should include data from longer periods such as 8 to 10 years^12,13^ to draw further conclusions about the effects of CON regulation after COVID-19. Additional analysis should also control for extraneous variables and changes within CON and non-CON states. Future studies could include private insurance payments in addition to Medicare to make the data more broadly representative of health care in the United States. Further analysis separating Arizona, Minnesota, and Wisconsin could help determine if this affects the observed trends and differences. Expansion of the cataract surgery codes analyzed, especially those for complex surgery, could add depth to the analysis. It would be impactful to study the cataract utilization trends in Texas and its respective counties, given its statistical influence in our study. It would also be interesting for further studies to examine the influence of state demographics, the timing of when the CON laws were put in place, and specific regulations the CON laws have on cataract surgery, as these factors may further influence the utilization and reimbursement rates of cataract surgery in a state-dependent manner.

## CONCLUSION

Healthcare costs, which constitute a significant portion of the US GDP, will continue to increase as our population ages. This study aimed to examine the impact of CON legislation on cataract surgery utilization and reimbursement. Regulations such as CON laws may have unintended consequences of limiting access to care, decreasing competition, and stifling needed growth. Surgeons should be aware of such regulations and their potential to impact not only individual providers but also the entire healthcare landscape. Further study into the economic and population health impacts is warranted to clarify CON laws’ role in the regulation of cataract surgery.

### Disclosures

The authors report no conflicting relationships.

## Supplementary Material

Online Supplementary Material
