# Supplementary material for: Examining the Impact of Certificate of Need Laws on the Utilization and Reimbursement of Cataract Surgeries Among Medicare Beneficiaries
Source: J Health Econ Outcomes Res. 2024 Aug 13;11(2):35–40. doi: 10.36469/001c.121618 (PMC11392485; doi:10.36469/001c.121618)
Supplement: Online Supplementary Material [file jheor_2024_11_2_121618_241495.pdf]

Journal of Health Economics and Outcomes Research

Online Supplementary Material

Examining the Impact of Certificate of Need Laws on the Utilization and Reimbursement of Cataract Surgeries Among Medicare Beneficiaries. *JHEOR*. 2024;11(2):35-40. doi:10.36469/jheor.2024.121618

Table S1: State Certificate of Need Status: 2017-2021

This supplementary material has been provided by the authors to give readers additional information about their work.

This is an open-access article distributed under the terms of the Creative Commons Attribution 4.0 International License (CCBY-4.0). View this license's legal deed at http://creativecommons.org/licenses/by/4.0 and legal code at http://creativecommons.org/licenses/by/4.0/legalcode for more information.

Table S1. State Certificate of Need Status: 2017-2021

**CON States**	**Non-CON States**
Alabama	Arizona
Alaska	California
Arkansas	Colorado
Connecticut	Idaho
Delaware	Kansas
Florida	Minnesota
Georgia	New Hampshire
Hawaii	New Mexico
Illinois	North Dakota
Indiana	Pennsylvania
Iowa	South Dakota
Kentucky	Texas
Louisiana	Utah
Maine	Wisconsin
Maryland	Wyoming
Massachusetts	
Michigan	
Mississippi	
Missouri	
Montana	
Nebraska	
Nevada	
New Jersey	
New York	
North Carolina	
Ohio	
Oklahoma	
Oregon	
Rhode Island	
South Carolina	
Tennessee	
Vermont	
Virginia	
Washington	
West Virginia	

Abbreviation: CON, certificate of need.
